# Educational Robotics and Tangible Devices for Promoting Computational Thinking

**DOI:** 10.3389/frobt.2021.713416

**Published:** 2021-11-15

**Authors:** Matthias G. Funk, Jose Manuel Cascalho, Ana Isabel Santos, Armando B. Mendes

**Affiliations:** ^1^ LIACC, FCT University of the Azores, Ponta Delgada, Portugal; ^2^ NICA, FCSH University of the Azores, Ponta Delgada, Portugal

**Keywords:** tangible programming languages, educational robotics, computational thinking, language complexity, human computer interaction

## Abstract

Recently, efforts have been made to add programming activities to the curriculum that promote computational thinking and foster 21st-century digital skills. One of the programming modalities is the use of Tangible Programming Languages (TPL), used in activities with 4+ year old children. In this review, we analyze solutions proposed for TPL in different contexts crossing them with non-TPL solutions, like Graphical Programming Languages (GPL). We start to characterize features of language interaction, their use, and what learning activities are associated with them. Then, in a diagram, we show a relation between the complexity of the languages with factors such as target age and output device types. We provide an analysis considering the type of input (*e.g.*, TPL *versus* GPL) and output devices (*e.g.*, physical robot *versus* graphical simulation) and evaluate their contribution to further insights about the general trends with respect to educational robotic systems. Finally, we discuss the opportunities to extend and improve TPLs based on the different solutions identified.

## 1 Introduction

Nowadays, the pervasiveness of technologies in people’s lives is unquestionable, particularly in children’s lives, from a very early age. This technology, whose use begins in the family, quickly extends to the school context, where we have tried to create spaces for its use in a meaningful way.

In the search for a child-centered implementation, which escapes a traditional educational framework, the introduction of educational robotics in school contexts seeks to provide children with the opportunity to research, discover, and apply knowledge in an authentic context (Somyürek, 2015). In this constructivist perspective of learning, it is intended, as Biggs (1996) points out, that students autonomously construct the meaning of their learning, more than having the meaning transmitted from teacher to students. In this text, educational robotics is understood as a set of activities designed to introduce students to robotics and programming in an interactive way from an early age. Learning and development does not happen alone but is built through the interactions that children have with their peers and with people from their immediate context (Vygotski, 1978).

There are many advantages to using robots in an educational context. In the classroom, the learning experiences intentionally designed by the teacher in educational robotics allow to provide space for children to solve problems cooperatively, through active experimentation, the use of language, explaining points of view, discussing, and analyzing the best solutions to solve problems (Jia, 2010). This type of pedagogical strategy based on a cooperative learning logic, implies shared learning experiences between all students but, especially, between the two genders. Studies point out the differences between the genders’ views and also the differences in how they interact with the technology, associating girls with more participatory activities (Manero et al., 2017; Kinzie and Joseph, 2008; Sauer et al., 2020). As indicated by Furtner et al. (2021), tasks with technology that involve problem-solving and, so, cooperation among the children, seems like an excellent strategy to promote girls’ involvement, “if they know that others contribute (more), they are more likely to contribute as well” (p.47). In this sense, applying this strategy using technology may contribute to reducing the gap between genders, a problem that has been tackled worldwide (García-Holgado et al., 2019). With the use of robots in the classroom many other skills are developed and valued in a significant way. In addition to the social aspects, the use of these devices allows the development of several technical and academic skills and have applicability with children with learning disabilities, as we will see below.

**FIGURE 1:**
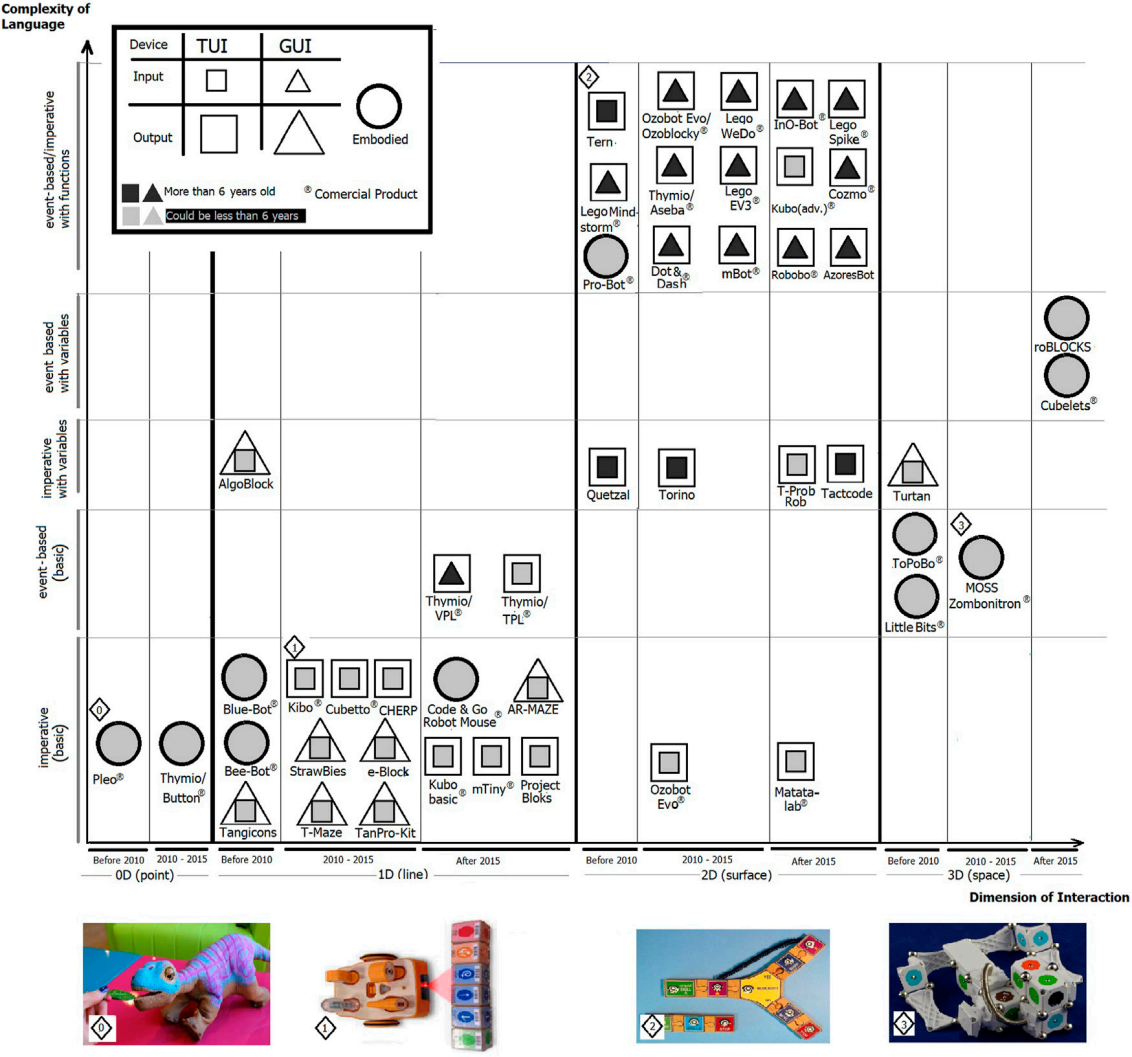
Classification of the tangible solutions based on input *versus* output devices, the complexity of programming language used and the year of their release. Bellow the diagram, pictures from 0 to 3 depict TPL examples, organized in the different space dimensions. From left to right, the dinosaur Pleo (0) interacts with touch, the Kibo (1) reads a sequence of commands, the Tern (2) represents the code in a space configuration and, finally, MOSS (3) uses a 3D space language in a mix activity of programming and robot building.

In this perspective of meaningful learning, and as an alternative to traditional programming interfaces, the manipulation of physical objects or tangible devices for programming is revealed as a fundamental learning strategy, increasing the range of ages that could be considered suitable for learning how to program. The concept of Tangible User Interfaces (TUI) was first introduced by Ishii and Ullmer (1997) as opposing to the Graphical User Interfaces (GUI). Therefore, the concept of TPL adapts the idea of “augment the real physical world by coupling digital information to everyday physical objects and environments” (idem) to the programming activity (see examples of these languages in Figure 1). The first experiments with TPL were created at the MIT Logo Lab in the mid-1970s controlling a floor turtle called TORTIS (Papavlasopoulou et al., 2017). Since then, a huge diversity of solutions emerged, addressing preschoolers with 4 or 5 years olds^1^.

TPL interfaces have been shown to increase active player engagement in informal learning Melcer and Isbister (2018). This more intuitive, playful and cooperative approach has the potential to provide shared learning experiences, overcoming the differences in the way both genders use the robots, as mentioned above.

Moreover, if learning is favored when the task includes the coordination of points of view and the exchange of ideas in a cooperative logic, the use of “things” (tangibles, matters, objects, artifacts, materials) in these collaborative activities is revealed as a facilitating strategy for the learning processes, serving as important mediators in this work logic (Heinemann et al., 2011). Making learning more manipulative, particularly with younger children, enables knowledge construction processes to take place involving the physical exploration of objects, particularly regarding the use of educational robotics (Sapounidis et al., 2015).

There are two other reasons usually addressed for those supporting TPL. The first is that Computer Thinking (CT) is an important learning outcome for programming activity where TPL apply better for younger children. The latter is that the use of TPL promotes active learning in classes fitting to new teaching methods such as Problem Based Learning or Project Based Learning (Valls et al., 2018) which are known as motivating and fostering active participation of children in classes.

In this sense, this paper seeks to offer an analysis of a wide range of existing systems with TPL. It seeks to understand the type of programming language they offer, the type of target audience they are aimed at, as well as the types of output devices, in an attempt in an attempt to better understand how current solutions seek to respond to the needs of meaningful, contextualized and relevant learning situations for children.

Along the review we apply the word *object* or, more generally, *system* to all solutions that have a tangible interface as an input or, in some cases, as an output. And we use *device* to identify each entity of these systems.

## 2 Dimensions Adopted for Bibliographic Revision

The bibliographic revision was made considering two main directions:

• To select TPL solutions that have impact at the moment they were presented (*e.g., Algoblocks* Suzuki and Kato, 1995) and that provide novelties in what might be considered the mainframe (*e.g., AR-MAZE* Jin et al., 2018).
• To include the maximum number of different solutions, in terms of robots and programming complexity (*e.g.,* identifying languages with and without function or variable concepts).

In Table 1 we compare different tangible solutions. We divide the dimensions presented in the first row into two categories. The first, technical, is related to the language of communication and several of its characteristics. The latter is related to different educational dimensions. The last column depicts the references to the pedagogical features associated with the language. The search was not exhaustive and its purpose was to find reports of the use of languages in educational contexts.

**TABLE 1:** Comparing different tangible solutions. Programming languages were defined as blocks with switches (BwS), buttons (Bts), sequence of commands board (Brd), surface sensors (SfS), paper tiles (Tls), colour strips (CStr)and embodied (E). The communication to the output device is hardwired (H), by Bluetooth (B), image processing (I), wireless (W), optical device (O), RFID (RFD) or embedded (E). The output device is a robot (R) or a screen (S), a screen with augmented reality (AR), an electronic board (eB) or an electronic blocks configuration (Bc). Technical and Academic Skills were identified as computational thinking (CT), logical (LT) and critical thinking (CrT), problem-solving skills (PS), using drawings for programming (Dr), ability to use and understand programming language (PL), sequencing (S), developing of multisensory (MsL) and sensorimotor (SmE) skills. At Social and Personal skills, collaborative learning (CL), negotiation (N), creativity (Cr), Cooperation and collaboration (Cp) motivation (M), communication (Cm), active participation (AP), playful or fun (PL) were found in the bibliography. Finally, for Learning disabilities were identified Autism Spectrum Disorders (A), children in hospitalization (H), Down Syndrome (D), special needs(SE) and visual disabilities (V).

	Language type	Year	Age	Emb. electronics	Communication	Multi-level (input)	Device type (output)	Low-cost system?	Pedagogical kit?	Tech. and acad. skills	Social and pers. skills	Learn. disabilities	—
Algoblock	BwS	1993	5–9	y	H	n	S	n	n	LT PS	CL	—	Suzuki and kato (1995)
Bee-bot© Pro-bot©	Bts	2000	4–6	Y	B	y	R	y	y	—	CL	A	Vázquez et al. (2019)
Blue-bot©	Bts Brd	2005	5+	y	B	y	R	y	y	—	CL	A	Bellegarde et al. (2019)
Pleo©^†^	SfS	2006	5+	y	B	y	R	y	y	Dr	Cr Cp	H	Moerman and Jansens. (2020), Ryokai et al. (2009)
QUETZAL	Bks	2007	12+	n	I	n	S/R	n	n	PS PL	Cp	—	Horn and Jacob (2006)
PROTEAS T_ProRob T_Butterfly	Tls	2011	6+	n	H/B	n	R	n	n	—	Cp Cr N	H	Sapounidis et al. (2015), Sapounidis et al. (2019)
CHERP	Bks	2012	4–6	N	W	n	R	y	n	CT PS S	—	—	Kazacoff et al. (2012)
E-BLOCK	Bks	2012	4–10	Y	W	n	S	y	n	MsL PS	CL Cp	SE	Wang et al. (2013)
Ozobot©	CStr	2012	6+	Y	O/B	n	R	n	y	CT PS	Cp M	—	Žáček and Smolka. (2019)
CUBETTO©	Brd	2013	3–9	N	B	n	R	n	y	PS S	Cp	—	Anzoátegui et al. (2017), Berson et al. (2019), Murcia et al. (2019)
Tanpro-Kit	Bks	2013	5–8	y	W	n	eB	n	n	PS	Cp	—	Wang et al. (2013)
Cublets©	E	2014	6–16	y	E	n	Bc	n	N	PS	Cp	A	Jung et al. (2020)
Kibo©	Bks	2014	4+	n	O	y	R	N	y	—	Cp	A D	Albo-Canals et al. (2018), González-González et al. (2019)
STRAWBieS	Tls	2015	5+	n	I	n	S	N	n	PS SmE	CL	—	Hu et al. (2015)
AR-MAZE	Bks	2018	5–9	n	I	n	S AR	Y	n	LT PS	—	—	Jin et al. (2018)
Tactcode	Tls	2018	6+	n		n	R	y	n	—	—	—	Cardoso et al. (2018)
Kubo©	Tls	2019	4+	y	RFD	y	R	N	y	CrT	Cp Cm Cr	—	Bertel et al. (2019)
Thymio TPL©	Tls	2019	4+	n	I	n	R	n	y	—	AP Cp PL	—	Mussati et al. (2019)
TORINO	E	2020	7–11	y	E	n	R	n	n	—	—	V	Morrison et al. (2020)

The technical dimensions selected identify the type of TPL, the year, the age range, if the TPL has or not embedded electronics, the output interface (*Device type*) and how the system communicates with it. It also indicates if the system provides more than one level, *i.e.,* if it is adaptable to users of other ages (*Multi-level*). Finally, it divides the systems into low-cost (*i.e*., if all proposed system components cost less than 100 euro) or non-low-cost solutions.

Concerning TPL, we see an impressive diversity, which correlates to the technical solutions provided by the languages used. Algoblock proposes the idea of using blocks that fit together in a programming sequence. That same idea is then used in E-BLOCK and also in Kibo. QUETZAL has adopted a similar strategy, but now the blocks are more like jigsaw puzzles because they attach together. This same idea is used in Tractcode and also in TPL Thymio’s version but now as tiles. The first column shows this diversity presenting different acronyms for each solution, from blocks (Bks) and tiles (Tls) to buttons or boards, filled with tiles instructions (Brd). Other solutions are particular to the type of output device used. Pleo, a robotic dinosaur, uses surface sensors (SfS), while Ozobot reads previous programmed colour strips codes (CStr).

In a nutshell, the Table 1 provides the additional following information:

• The use of TPL extends classes programming activity for the range of 5+ years or even 4+ years old.
• First TPL adopted embedded electronics because of the difficulty of using another type of communication at the time.
• TPL are also used to control virtual entities in simulation graphical environments (*e.g.,* AR-MAZE or PROTEAS).
• All comercial TPL (with ©) are available^2^.

In educational terms, the tangible objects analyzed can be considered from three major dimensions or analysis categories, according to the skills they seek children to develop and their functionality as a tool to facilitate learning in children with learning disabilities. Thus, we found objects clearly directed towards the development of technical and academic skills, focused on aspects related to the nature of programming tasks and the concepts associated with them, as well as academic learning, is inherently enhanced through their use. These technical and academic skills, present in practically all the objects targeted for analysis, have to do with , for example, the understanding and mobilization of concepts in the construction of Computational Thinking (CT^3^) (Sáez-López et al., 2019; Uşengül and Bahçeci, 2020; Žáček and Smolka, 2019), the ability to use and understand the programming language (PL) (Horn and Jacob, 2006; Uşengül and Bahçeci, 2020; Žáček and Smolka, 2019; Riedo et al., 2013), sequencing (S) (Anzoátegui et al., 2017; Berson et al., 2019), problem-solving skills (PS) (Sáez-López et al., 2019; Jin et al., 2018; Jung et al., 2020; Sapounidis et al., 2015; Wang et al., 2013), the ability to develop logical (LT) (Jin et al., 2018) and critical thinking (CrT) (Bertel et al., 2020), or the development of multisensory (MsL) (Wang et al., 2013) and sensorimotor (SmE) skills (Hu et al., 2015). Finally, there is one reference to programming using drawings (Dr)(Moerman and Jansens, 2020).

In parallel, in several objects it is possible to identify a set of personal and social competencies that are important in the learning process that take place simultaneously with the development of technical and academic competencies. The development of these competencies is related to the creation of collaborative spaces that encourage interaction between students. At the social level, most of these competencies relate to cooperation or collaboration (Cp) which is promoted with the implementation of group programming activities and the exchange of experiences (Albó-Canals et al., 2018; Heljakka and Ihamäki, 2019; Jung et al., 2020), but also to collaborative learning (CL) which takes place in these types of activities (Pérez-Vázquez et al., 2019) and negotiation (N) (Sapounidis et al., 2019; Sapounidis et al., 2015). The use of tangible objects also allows the development of a set of personal aspects such as creativity (Cr) (Bertel et al., 2020; Moerman and Jansens, 2020; Riedo et al., 2013; Ryokai et al., 2009), motivation (M), communication (Cm) (Bertel et al., 2020), the playfulness or fun they promote (PL)(Sáez-López et al., 2019; Sapounidis et al., 2015; Sapounidis et al., 2019) or allowing the child to have active participation in the process (AP) (Mussati et al., 2019).

We also identified objects that, although they can be used by any child, have applicability with learning disabled children, in particular children with Down Syndrome (D) (Albó-Canals et al., 2018), Autism Spectrum Disorders (A) (Jung et al., 2020; Pérez-Vázquez et al., 2019; González-González et al., 2019), special needs (SE) (Bertel et al., 2019) children in hospitalization (H) (Moerman and Jansens, 2020), and specifically with children with visual disabilities (V) (Morrison et al., 2020). In the table, a system is considered a pedagogical kit when, along with it, a set of resources (*e.g.,* manuals, exercises adapted to specific school years) can be acquired by the user. Usually, a multi-level approach comports the use of a pedagogical kit but not necessary the other way around.

Finally, the objects can demonstrate their longevity. Pleo, thanks to its open-code facility, allowed new experiences transforming the way children could interact with it (Moerman and Jansens, 2020).

## 3 Programming Using Tangible Devices

To characterize programming languages, we organize seven different dimensions associated to each object, as depicted in the diagram in Figure 1, as follows:

• *x-axis* identifies haptic user interface dimensions associated with programming activities;
• *y-axis* divides language complexity into basic imperative or event-based languages (*i.e*., without the use of variables), imperative or event-based (with the use of variables) and languages with the use of variable and functions;
• Each object has its release year divided into three intervals, *i.e*., those released before 2010, those released between 2010 and 2015 and those released after 2015;
• The objects are divided into two groups based on the minimum age indicated by the authors or researchers who tested the device, *i.e*., suitable for children under 6 years old (pre-school children) *versus* over 6 years old;
• Each object is characterized by its input and output type: a GUI, TUI or an embedded device and;
• Each object is identified if it is a commercial product.

The first feature to notice in the diagram is the representation of the input *versus* output devices using squares and triangles to distinguish tangible solutions from graphical ones. As a support to this representation, we use the more general concepts of TUI and GUI to distinguish the devices used by each system for programming and executing activities. For example, T-Maze input device is a TUI type (square) because it uses a TPL for programming while the screen output device is a GUI type (triangle). Thymio VPL input device is a GUI (triangle) using a VPL for programming and the output device is TUI type (square), using a robot. The embedded interface types correspond to the cases that a tangible object works both as an input and output device. A paradigmatic example are the popular Bee-bot and Blue-bot robots. This classification allowed us to include non-TPL solutions (*e.g.,* Lego or mBot) and they were added to extend the analysis to the language dimension complexity.

As the haptic sense is directly related to space, we use the spatial metaphor to classify input and output device interaction. The simplest is a punctual interaction, extended into a linear when a sequence of instructions controls the output. We call planar interaction adding alternatives or loops, as it could be represented as a path in a plain. If the input and output devices are embedded, this interaction could be driven by the pathway of their spatial topology configuration, which we see as a 3D interaction. In general, this last type of interaction includes hardware (or embodied) programming where the robot’s behavior is defined by the embedded sensors and actuators, like in Cubelets.

Following the horizontal axis direction from left to right, we can classify the proposed systems based on the complexity of the used language. Furthermore, following from bottom to top, one can identify the physical dimension of the language used. One highlight is that objects can have many types of input interactions centered on a specific robot device. As an example, the Thymio objects are presented in the figure with four different configurations. At the left, Thymio/button robot responds directly to the touch of a button. Then, in the 1D linear interaction, the Thymio is programmed by GPL or TPL (or paper-code) languages. Finally, using Aseba language, which uses loops and alternatives, the same robot moves into the planar interaction increasing the programming complexity.

In summary, we can identify the trends that are explicit in the diagram as follows:

• As expected, the top right side of the diagram concentrates most objects with TPLs. In contrast, in the bottom left, the solutions are mostly the GPL associated with specific robots;
• The solutions with more simple languages are also used by the youngest children with the darkest solutions at the bottom left in the diagram with few exceptions such as Kubo;
• Along the years there is a significant increase of commercial products available, possibly due to the sprawl of the importance of Computational Thinking and the maturity of solutions found.

Digging into the details of the solutions proposed, we found trends that could be detected but that somehow are not directly visible in the diagram. They are:

• New objects can use Artificial Intelligence (AI) in their conception or apply AI algorithms in their behaviors (*e.g., COZMO*, *Robobo*);
• Some systems can offer effective complex TPL (*e.g., Kubo*);
• Consolidated solutions tend to adopt languages with increasing complexity to provide solutions adapted to different ages (*e.g.*, *Thymio*);
• Several solutions explore sensory interaction methods, using touch or sound to program (*e.g., TORINO*, *Pleo*);
• New objects improve their “social connection” by improving their empathy to users (*e.g.,* Dash and Dot, *COZMO*).

## 4 Conclusion

In this review, we identify a considerable diversity of TPL solutions analyzed in different dimensions and following two different approaches. In the first, we explore technical issues and how researchers see their use in educational environments, in Table 1. In the latter, a diagram characteries the type of interfaces, extending the objects to TPL and GPL interaction types, and how solutions spread in haptic and language complexity dimensions, in Figure 1. As expected, TPL addresses mainly the solutions with languages with less complexity and the number of recent commercial products shows the increasing interest in this type of interaction. The diversity of solutions is visible by the number of different input devices and how they communicate to the output devices as well as by the dimensions of interactions provided.

In educational terms, this analysis demonstrates, on the one hand, the relevance of tangible objects to educational processes and spaces that are increasingly useful in defining strategies that seek their development in a more meaningful, active and interesting way for children and young people. All of them are focused on the acquisition of skills related to programming and computational thinking at different levels of complexity. However, many of them go beyond those mere technical skills, enhancing social interactions, collaborative learning, communication, playfulness, problem-solving and the promotion of learning in specific areas of knowledge. The use of these objects for special educational purposes is also interesting, contributing to more inclusive education.

However, this focus on developing programming skills and other learning associated with them for children and young people’s academic and professional development is beginning to prove, to some extent, reductive or simplistic. Bers (2019) draws attention to the need to look at computer science in schools, particularly from early childhood education, as a tool for the training of future active and participatory citizens and to understand programming as another form of literacy, close to the emergent perspective of Ferreiro and Teberosky (1996) or Sulzby (1985), proposing a new way of approaching computer science in early childhood education called “Coding as Another Language” (CAL). In any case, it is about bringing the way programming happens closer to the way young children think about written language, contributing to the construction of thinking about languages, regardless of their nature, an aspect that should deserve particular attention in future research in this area, due to its relevance in terms of approaching aspects of child development.

Finally, this analysis provided insights related to recent proposals and highlights inevitably exciting novelties in the following years. One is the combined used of TPL with augmented reality (*e.g., AR-MAZE*), allowing young children to program tangibly and test their code in an environment with additional support, such as debugging tools. Another is the embodied code interpreted solution (*e.g., Kubo*), where robots read and interpret code, giving feedback about possible codifying errors. This solution seems to bring robots closer to its users by including them in the programming task, and enhancing its social interaction role. Also, with the increasing technology complexity, more solutions provide sophisticated behaviors in robots adding new valencies that extend communication capabilities, enlarging the target of children with learning disabilities and transforming the way children communicate with the robotic systems.

## Footnotes

1 In contrast to TPL, block based programming languages used in a Graphical User Interface will appear in the text as Visual Programming Language (VPL) or Graphical Programming Language (GPL).

2 Note that Moerman and Jansens (2020) uses a modified version of Pleo software.

3 See Table 1 to associate to the object.
